# Effects on heavy menstrual bleeding and pregnancy of uterine artery embolization (UAE) or myomectomy for women with uterine fibroids wishing to avoid hysterectomy: The FEMME randomized controlled trial

**DOI:** 10.1002/ijgo.14626

**Published:** 2023-01-17

**Authors:** Fusun Sirkeci, John Moss, Anna M. Belli, Klim McPherson, Jane Daniels, Isaac Manyonda, Lee Middleton, Versha Cheed, Olivia Wu, Mary A. Lumsden, the FEMME Trial Collaborative Group

**Affiliations:** ^1^ Department of Obstetrics and Gynaecology St Mary's Hospital, Manchester NHS Foundation Trust Manchester UK; ^2^ School of Medicine University of Glasgow Glasgow UK; ^3^ Department of Radiology St George's Hospital and Medical School London UK; ^4^ Department of Primary Care University of Oxford Oxford UK; ^5^ Nottingham Clinical Trials Unit University of Nottingham Nottingham UK; ^6^ Department of Gynaecology St George's Hospital and Medical School London UK; ^7^ Birmingham Clinical Trials Unit University of Birmingham Birmingham UK; ^8^ Institute of Health and Wellbeing University of Glasgow Glasgow UK

**Keywords:** female, myomectomy, ovarian reserve, pregnancy rate, quality of life, United Kingdom, uterine artery embolization, uterine fibroid

## Abstract

**Objective:**

To determine treatment options (myomectomy vs. uterine artery embolization (UAE)) for women wishing to avoid hysterectomy.

**Methods:**

A multicenter randomized controlled trial was conducted on 254 women and data were collected on fibroid‐specific quality of life (UFS‐QOL), loss of menstrual blood, and pregnancy.

**Results:**

At 4 years, the mean difference in the UFS‐QOL was 5.0 points (95% confidence interval (CI) −1.4 to 11.5; *P* = 0.13) in favor of myomectomy. This was not statistically significant as it was at 2 years. There were no differences in bleeding scores, rates of amenorrhea, or heavy bleeding. Of those who were still menstruating, the majority reported regular or fairly regular periods: 36 of 48 (75%) in the UAE group and 30 of 39 (77%) in the myomectomy group. Twelve women after UAE and six women after myomectomy became pregnant (4 years) with seven and five live births, respectively (hazard ratio 0.48, 95% CI 0.18–1.28). There was no difference between the levels of hormones associated with the uterine reserve in each group.

**Conclusion:**

Leiomyoma are common in reproductive‐aged women, causing heavy menses and subfertility. Among women with uterine fibroids, myomectomy resulted in better fibroid‐related quality of life at 4 years, compared with UAE but the treatments decreased menstrual bleeding equally. There was also no significant difference in the impact of treatment on ovarian reserve.

## Synopsis

Women with uterine fibroids were randomized between uterine artery embolization and myomectomy. Quality of life was greater in the myomectomy group at 2 years, but neither menstrual blood loss nor markers of ovarian function differed.

## 1 INTRODUCTION

Uterine fibroids are the most common tumor in women of reproductive age, with increasing prevalence as women get older. It is estimated that by the time they are in their 50s, around 80% of women will have developed a fibroid.^1^ Fibroids may have significant symptoms such as heavy menstrual bleeding and a feeling of pressure, which can affect the patient's quality of life.

Recently, childbirth trends have shifted, and more women appear to delay starting their families until their third or fourth decade.^2^ These are the ages when fibroids are more common and symptomatic. Fibroids have a higher prevalence in African American women, signifying a marked disparity in health needs when compared with white women.^3^ This trend may have driven an increased demand for uterine‐saving fibroid treatments.^4^ This change in demographics, coupled with the increased availability of newer treatment options, warrants that patients should be advised on the best evidence‐based treatment.

The symptoms experienced by women with fibroids may vary depending on the position, size, and number of fibroids. Intramural fibroids are the most common form of fibroid but are frequently asymptomatic. Fibroids can become very large and are often associated with heavy menstrual bleeding. Some clinicians believe that the presence of fibroids may have a negative impact on fertility^5^, ^6^ although the data are contradictory.^7^

There is still uncertainty as to which is the best treatment for a woman with symptomatic fibroids who wishes to preserve her uterus. To address this, existing and new treatments should be fully evaluated from all perspectives, including the relief of symptoms and the impact on the woman's quality of life, and the data should be subject to careful review.^8^ Surgery, either myomectomy or hysterectomy, has traditionally been the main approach for the management of symptomatic fibroids. Myomectomy, while preserving the uterus, can lead to myometrial trauma. Myomectomy can be undertaken laparoscopically, hysteroscopically, or by a laparotomy.

Uterine artery embolization (UAE) involves the temporary occlusion of the arteries supplying the uterus using biocompatible particles and is usually performed under local anesthetic. It causes ischemic infarction from which the uterus usually recovers but the fibroids do not. The use of UAE in women who may wish to conceive is controversial within some sections of the medical community.^9^

Both myomectomy and UAE appear to improve the quality of life of women with symptomatic uterine fibroids, but data from high‐quality studies are sparse. For the control of symptoms, the choice is currently unclear and indications and clinical preferences for either modality are varied. Perhaps as a result of the uncertainty of the effect of UAE on fertility, previous randomized controlled trials (RCTs) have not generally included women wishing to get pregnant. This meant that the studies undertaken are poorly designed and inconclusive. One small study reported that the women in the myomectomy arm became pregnant sooner postoperatively than the women in the UAE arm, and live birth rates were significantly lower among the UAE group (relative risk (RR) 2.32, 95% confidence interval (CI) 1.19–4.53; *P* = 0.01).^10^ These results and concerns around the potential impact of UAE on ovarian and uterine function have resulted in recommendations against UAE for women seeking pregnancy^9^ as the impact on mean serum concentrations of anti‐Müllerian hormone (AMH) and follicle‐stimulating hormone (FSH) was uncertain.

UAE has been performed in Africa and Asia, with reports of success even with large fibroids, as are common in Africa.^3^ There are several reports of good outcomes such as would be anticipated elsewhere^11^, ^12^ and, overall, its introduction has been a positive experience for many women in both Africa and Asia.^13^

Myomectomy is also undertaken, but the literature reports a reluctance due to fear of uterine rupture in this and future pregnancies.^14^, ^15^ In addition, the incidence of other complications, such as deep vein thrombosis, may differ from that in western countries.^14^

When compared with the open route, the safety and efficacy of laparoscopic myomectomy^16^ may make it the optimum choice for those with the necessary skill.

Papers discussing UAE are often not in the gynecological literature, which is perhaps not surprising as UAE is undertaken by an interventional radiologist, and many gynecologists around the world are protective of their practice and prefer to manage women from diagnosis to recovery.

The aim of the present multicenter open RCT was to assess the clinical effectiveness of these two uterine‐saving fibroid treatments.

## 2 MATERIALS AND METHODS

The present study is an open, randomized, parallel multicenter trial comparing two uterine‐saving fibroid treatments with ethics approval from the Coventry and Warwickshire National Research Ethics Service Committee (Reference: 11/WM/0149). It was approved by the UK National Research Ethics Service and the research department at each participating hospital.

The participants were recruited from 29 participating hospitals across the UK. Following a full clinical assessment and imaging to confirm the presence of fibroids, the patients were invited to the study if the assessing clinician felt the patient would benefit from either myomectomy or UAE. A trial‐approved member of the local team would then approach the patient at a later date and provide written information on the study. Patients who consented to take part were given a baseline questionnaire. The majority of the participants had magnetic resonance imaging (MRI) scans to map the fibroids. Patients were randomized if they met all the inclusion criteria and none of the exclusion criteria.

The inclusion criteria were as follows: women with symptomatic fibroids who did not wish to have a hysterectomy, but who were prepared to accept one in an emergency; women suitable for, and accepting of, either myomectomy or UAE; women for whom the clinical team were uncertain as to which treatment was indicated; and women who provided written informed consent.

The exclusion criteria were as follows: women who refused a hysterectomy, even if an intraoperative complication made this an advisable procedure; women with recent or ongoing pelvic inflammatory disease; women with significant adenomyosis, as identified by transvaginal ultrasound or MRI (women with concurrent adenomyosis were eligible if fibroids were believed to be the predominant cause of their symptoms); women with a positive pregnancy test just before consent; postmenopausal women, defined as more than 1 year since the last menstrual period; women with suspected malignancy; women aged under 18 years; women who were unable to provide informed consent because of incapacity (as defined by the Mental Capacity Act 200555 or the Adults with Incapacity (Scotland) Act 200056); non‐English‐speaking women for whom translation or interpretation facilities were insufficient to guarantee informed consent; and women who had previously undergone myomectomy via a laparotomy or had previously undergone embolization.

A sample size of 250 participants was estimated to have a power of 90% (at a two‐sided alpha level of 0.05) to detect a moderate‐sized difference between the groups (i.e., 0.55 of a standard deviation in the primary outcome).

The analysis of the primary outcome was performed in accordance with the intention‐to‐treat (ITT) principle. Statistical analyses were computed on complete observed data and all randomized participants at all assessment times through the imputation of missing responses. Repeated‐measures linear regression models, including data at all time points, were used to estimate least‐square mean differences (with 95% two‐sided CIs) in the primary outcome at 2 years. The model included participant, treatment group, baseline score, time, interaction between time and treatment group, and the minimization variables. Participants were included in the complete case analysis if they had at least one response at any of the three assessment time points. In the analysis that took missing responses into account, multiple imputation was performed with the use of the Markov chain Monte Carlo method, which assumed a joint multivariate normal distribution. The imputation model was consistent with the analysis model.^17^

For the primary outcome, a *P* value was obtained using a linear regression model. Observed data from secondary continuous outcomes were analyzed in a similar manner to the primary outcome. The levels of reproductive hormones were log‐transformed and were presented as geometric mean ratios. Log‐binomial regression was used to estimate relative rates and 95% CIs for binary outcomes, making similar adjustments to the other analyses. The widths of the CIs were not adjusted for multiplicity and, therefore, the intervals should not be used to infer definitive treatment effects.

Several sensitivity analyses for the primary outcome were also performed, including the following: the inclusion of time as a continuous linear predictor, assuming no interaction with treatment; the addition of a parameter for the treating hospital; and a per‐protocol analysis, including only those who received the allocated treatment. Some questionnaires were incomplete and, therefore, an additional sensitivity analysis used available subscale scores to generate an overall score.

For the 4‐year data, continuous outcomes were analyzed by adding responses at this time point to the aforementioned regression models. For time to first pregnancy and time to first further procedure for treatment of fibroids, a Cox proportional hazard model was carried out, adjusting for the minimization variables. Kaplan–Meier plots were produced, in which women were censored if they had withdrawn, were lost to follow‐up, or had undergone a hysterectomy.

The treatment effect on the primary outcome in pre‐specified subgroups that matched the minimization variables were analyzed involving adding the subgroup‐by‐treatment group interaction parameters to the linear regression model. All analyses were performed using SAS software version 9.4 (SAS Institute, Inc.,).

Participants were randomized to undergo myomectomy or UAE in a 1:1 ratio. The stratification variables were used for the minimization details of the fibroids and whether the woman desired pregnancy. The demographic and clinical criteria were requested for each randomized participant. These procedures were performed according to the local guidelines and clinician's preference.

The primary outcome was participant‐reported health‐related quality of life domain of the Uterine Fibroid Symptom and Quality of Life (UFS‐QOL) tool, which combines the assessment of symptoms as well as quality of life.^18^ The health‐related QOL score at 2 years of follow‐up was the primary time point. The data have been reported elsewhere.^17^, ^19^ The primary analyses were by ITT. A further follow‐up to 4 years was undertaken in a subgroup of women.

Blood tests were obtained at baseline, along with various demographic and clinical criteria. Patients were allocated to treatment, either myomectomy or UAE, following the randomization process. These procedures were performed according to the local guidelines and clinician's preference.

Secondary outcomes included loss of menstrual blood using the Pictorial Bleeding Assessment Chart (PBAC), pregnancy and related outcomes, patient acceptability, length of hospital stay, further treatments, assessment of ovarian reserve by measuring FSH, AMH, luteinizing hormone, serious adverse events, and complications.

### 2.1 Assessment of menstrual blood loss

The loss of menstrual blood was measured using the PBAC.^20^ Scores ranged from 0 (no bleeding) as a minimum but had no fixed upper limit. This is a validated and well‐used assessment of the loss of menstrual blood in women with uterine fibroids. A score of 100 is usually considered to be excessively heavy bleeding.^20^

### 2.2 Pregnancy outcomes

Pregnancy outcomes were recorded as specifically pregnancy (overall and in the population desiring pregnancy at the time of randomization) and outcomes (live birth, miscarriage, stillbirth, and termination).

### 2.3 Ovarian reserve

The ovarian reserve was measured by an assay of FSH, AMH, and luteinizing hormone. Hormonal levels were measured at baseline, 6 weeks, 6 months, and 1 year after the initial procedure. Blood samples were taken on day 2, 3, or 4 of the menstrual cycle.

**FIGURE 1:**
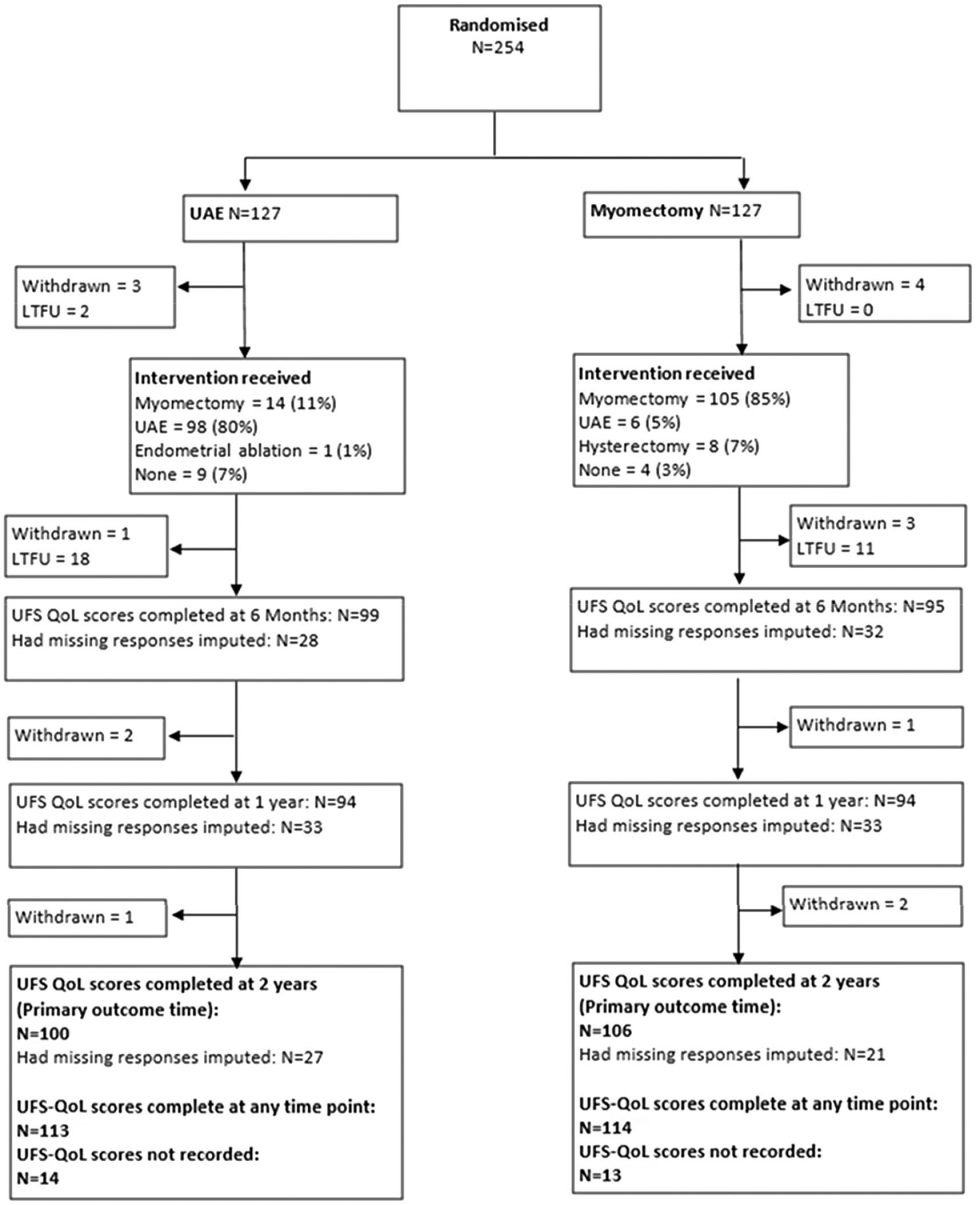
Flow of participants through the FEMME trial up to 2 years of follow‐up. LTFU, lost to follow‐up; UAE, uterine artery embolization; UFS‐QOL, Uterine Fibroid Symptom Quality of Life.

A total of 127 participants were assigned to the myomectomy group and 127 to the UAE group (Figure 1). The follow‐up rate for the primary outcome was 206 of 254 (81%) at 2 years, and 227 women (89%) provided scores at one assessment time at least.

## 3 RESULTS

The mean age of the patients was 41 years and they had a mean body mass index (BMI, calculated as weight in kilograms divided by the square of height in meters) of 28. There were no significant differences between the two groups with regard to demographics and in the number of women desiring pregnancy at randomization (UAE: 61 [48%]; myomectomy: 61 [48%]).

The baseline characteristics of the trial participants are shown in Table 1.

**TABLE 1:** The baseline characteristics of the trial participants^a^

Baseline characteristic	UAE group (*n* = 127)	Myomectomy group (*n* = 127)
Demographics and obstetric history		
Age (years)	40.2 ± 6.55	42.7 ± 6.4
Ethnic group		
White (British/other)	59 (46)	57 (45)
Black (Caribbean/African/other)	48 (38)	54 (43)
South Asian (Indian/Pakistani/Bangladeshi)	10 (8)	5 (4)
Mixed (white/black/Asian/other)	6 (5)	8 (6)
Other	4 (3)	3 (2)
BMI (kg/m^2^)	28.2 ± 6.2 (*n* = 119)	28.1 ± 5.3 (=123)
Desiring pregnancy at time of randomization^b^	61 (48)	61 (48)
Parity	0 (0–1) (*n* = 125)	1 (0–2) (*n* = 127)
Gravidity	1 (0–2) (*n* = 125)	2 (0–3) (*n* = 127)
Fibroid assessment		
Imaging modality to diagnose fibroid^c^		
MRI	89 (70)	99 (78)
Ultrasound	36 (28)	27 (21)
Not stated	2 (2)	1 (1)
Location of largest fibroid		
Submucosal	6 (5)	14 (11)
Submucosal (pedunculated)	1 (1)	1 (1)
Subserosal	30 (24)	21 (17)
Subserosal (pedunculated)	6 (5)	5 (4)
Intramural	74 (58)	81 (64)
Other	4 (3)	0
Not stated	6 (5)	5 (4)
Longest dimension of largest fibroid (cm)^b^		
≤7	64 (50)	64 (50)
>7	63 (50)	63 (50)
Mean ± SD	7.6 (3.2)	7.7 (4.2)
No. of fibroids +b		
1–3	84 (66)	84 (66)
4–10	37 (29)	37 (29)
>10	6 (5)	6 (5)
Median (IQR)	2 (1–5)	2 (1–5)
Largest fibroid volume (cm^3^)	436 ± 594 (*n* = 124)	446 ± 548 (*n* = 126)
Uterine volume (cm^3^)	1170 ± 1280 (*n* = 118)	1240 ± 1120 (*n* = 118)
Medical and surgical history		
Previous abdominal surgery^d^		
Cesarean delivery	12 (9)	19 (15)
Laparoscopy	19 (15)	15 (12)
Endometrial ablation	3 (2)	2 (2)
Appendectomy	8 (6)	7 (6)
Sterilization	4 (3)	5 (4)
Other	10 (8)	15 (12)
Taking contraceptive/hormonal treatments to control symptoms at randomization	75 (59)	73 (57)

Abbreviations: BMI, body mass index; IQR, interquartile range; MRI, magnetic resonance imaging; SD, standard deviation; UAE, uterine artery embolization.

^a^ Values are given as number (percentage), mean ± SD, or median (IQR).

^b^ Minimization variable.

^c^ More than one type of scan possible.

^d^ More than one previous abdominal surgery possible.

A substantial number of participants (38% in the UAE group and 43% in the myomectomy group) were of African Caribbean ethnicity and presented with a wide range of fibroid diagnoses. The rates of procedural complications were low in both groups, perhaps reflecting the expertise in the participating centers.

### 3.1 Quality of life

Myomectomy resulted in a greater increase in the quality of life at 2 years, although improvement occurred in both groups (mean difference from observed data: 8.0 points, 95% CI 1.8–14.1; *P* = 0.01; mean adjusted difference with missing responses imputed: 6.5 points, 95% CI 1.1–11.9 points). This has been reported in detail elsewhere.^17^, ^18^

### 3.2 Menstrual bleeding outcomes at 2 and 4 years

There were no apparent differences between the groups in terms of menstrual regularity. There were regular or fairly regular cycles in 88% of the UAE group and 61% of the myomectomy group, and the difference was not statistically significant.

There were no apparent and sustained differences in the bleeding scores, nor in the proportions of women reporting amenorrhea or heavy bleeding, between the two groups over the 2 years of follow‐up (Table 2). This was maintained at 4 years (Table 3).

**TABLE 2:** Pictorial Blood Assessment Chart bleeding scores and categories within the first 2 years

	UAE	Myomectomy	Estimated mean difference or relative risk (95% CI)^a^
Baseline (*n*)	102	100	
Total score^b^	133 (63–275)	180 (100–383)	
Amenorrhea^c^	0	1 (1)	
Light bleeding	0	1 (1)	
Normal	40 (39)	23 (23)	
Heavy^d^	62 (62)	75 (75)	
2 years (*n*)	75	77	
Total score^b^	32 (0–88)	41 (11–84)	
Amenorrhea^c^	19 (25)	14 (18)	0.7 (0.4–1.3)^e^
Light bleeding	5 (7)	5(6)	
Normal	36 (48)	41 (53)	
Heavy^d^	15 (20)	17 (22)	1.0 (0.5–1.8)^e^

Abbreviations: CI, confidence interval; IQR, interquartile range; SD, standard deviation; UAE, uterine artery embolization.

^a^ Estimates adjusted for baseline value and minimization variables.

^b^ Scores ranged from 0 (no bleeding) to ∞ (worst bleeding); differences of <0 favor myomectomy.

^c^ Relative risk for amenorrhea; estimates of >1 favor myomectomy.

^d^ Relative risk for heavy bleeding; estimates of <1 favor myomectomy.

^e^ Unadjusted model due to non‐convergence in adjusted model.

**TABLE 3:** PBAC bleeding scores and categories within 4 years^a^

PBAC score or category	UAE	Myomectomy	
Total score	28 (0–75)	29 (0–81)	
			RR (95% CI); *P* value
Amenorrhea (=0)	14 (27)	15 (35)	1.3 (0.7–2.3)^a^; 0.44
Normal (>10–100)	26 (51)	21 (49)	
Heavy (>100)	8 (16)	6 (14)	0.9 (0.4–2.4); 0.88
Total	51	43	

Abbreviations: CI, confidence interval; IQR, interquartile range; PBAC, Pictorial Blood Assessment Chart; RR, relative risk; UAE, uterine artery embolization.

^a^ Unadjusted model used because adjusted model failed to converge.

### 3.3 Pregnancy outcomes

There were 22 pregnancies in total: 15 in the UAE group and 7 in the myomectomy group. Of the 14 women who reported pregnancies within 2 years of randomization, nine were in the UAE group and five were in the myomectomy group, representing 17% and 10% of participants, respectively, who expressed a desire for pregnancy at the time of randomization. Of these, there were six and four live births, respectively, and two miscarriages in the group allocated to UAE (Table 4). These numbers were too small to draw any conclusions on the effect of the procedures on fertility.

**TABLE 4:** Pregnancy outcomes within 2 years^a^

	UAE	Myomectomy	RR (95% CI)^b^
Women reporting pregnancy	9/112 (8)^c^	5/112 (4)	0.6 (0.2–1.7)
Pregnancy (in women desiring pregnancy at time of randomization)	9/52 (17)	5/48 (10)	
Pregnancy outcome			
Live birth	6/106 (6)	4/107 (4)	–
Miscarriage^c^	2/106 (2)	0/107 (−)	–
Termination of pregnancy	1/106 (1)	1/107 (1)	–

Abbreviations: CI, confidence interval; RR, relative risk; UAE, uterine artery embolization.

^a^ Values are given as number (percentage) unless otherwise indicated.

^b^ Estimates >1 favor myomectomy.

^c^ One woman had two pregnancies that both ended in miscarriage and these have been reported once.

### 3.4 Pregnancy outcomes at 4 years

**FIGURE 2:**
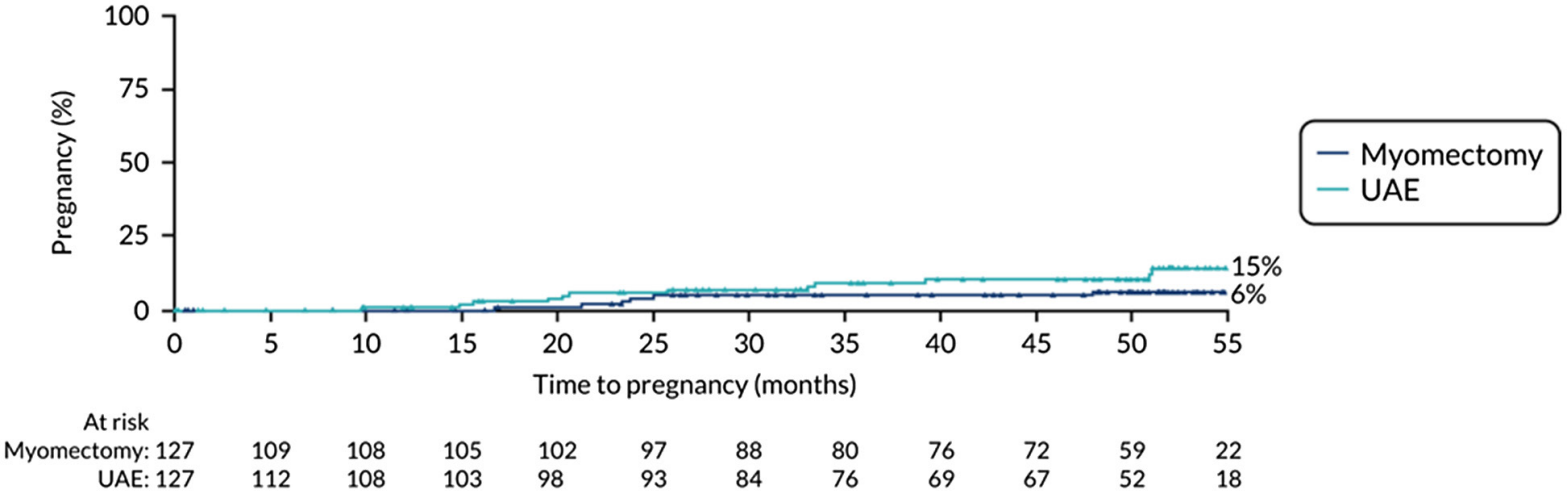
Kaplan–Meier plot of time to achieve pregnancy in the two groups who were followed up beyond the end of the study to 4 years. It indicates that numbers overall were low over that time. The numbers in the UAE group continued to rise. UAE, uterine artery embolization.

The number of women becoming pregnant are reported as cumulative rates in Table 5. The time to first pregnancy was plotted in a Kaplan–Meier curve (Figure 2). The cumulative pregnancy rate was 15% in the UAE group and 6% in the myomectomy group, giving a hazard ratio of 0.48 (95% CI 0.18–1.28; *P* = 0.14) (Table 6).

**TABLE 5:** Pregnancy outcomes at 4 years^a^

Outcome	UAE group	Myomectomy group
Pregnancy by ITT		
Women reporting pregnancy^b^	12 (15)	6 (7)
Pregnancy (in population desiring pregnancy at time of randomization)	12 (15)	6 (7)
Live birth	7 (9)	5 (6)
Miscarriage	4 (5)	0
Termination	1	1
Pregnancy by per protocol		
Women reporting pregnancy^c^	7 (8)	6 (7)
Live birth	4 (5)	5 (6)
Miscarriage	2	0
Termination	1	1
Pregnancy by treatment received		
Women reporting pregnancy^d^	7 (8)	8 (10)
Live birth	4 (5)	6 (7)
Miscarriage	2	1 (2)
Termination	1	1

Abbreviations: ITT, intention to treat; UAE, uterine artery embolization.

^a^ Values are given as number of women (number of events).

^b^ UAE group: one participant had two pregnancies that both ended in miscarriage and two participants had two pregnancies that both ended in live birth. Myomectomy group: one participant had two pregnancies that both ended in live birth. These events have been primarily included once in this table, with repeat events in the same woman shown in brackets. All other events occurred in separate women. Percentages of the total population cannot be calculated, as women withdrew from the trial or were lost to follow‐up at different intervals up to 4 years.

^c^ UAE group: one participant had two pregnancies that both ended in live birth. Myomectomy group: one participant had two pregnancies that both ended in live birth. These events have been primarily included once in this table, with repeat events in the same woman shown in brackets. All other events occurred in separate women. Percentages of the total population cannot be calculated, as women withdrew from the trial or were lost to follow‐up at different intervals up to 4 years.

^d^ UAE group: one participant had two pregnancies that both ended in live birth. Myomectomy group: one participant had two pregnancies that both ended in live birth and one participant had two pregnancies that both ended in miscarriage. These events have been primarily included once in this table, with repeat events in the same woman shown in brackets. All other events occurred in separate women. Percentages of the total population cannot be calculated, as women withdrew from the trial or were lost to follow‐up at different intervals up to 4 years.

**TABLE 6:** Hormonal markers of ovarian reserve over the 2 years of the study with no significant difference between the two groups^a^

	UAE	Myomectomy	Estimated geometric mean ratio, adjusted for baseline^b^ (95% CI)	Estimated geometric mean ratio, adjusted for baseline and age^b^ (95% CI)
AMH (pmol/L)				
Baseline	0.70 (0.52–0.94), *n* = 122	0.40 (0.29–0.56), *n* = 123		
6 weeks	0.45 (0.31–0.63), *n* = 90	0.26 (0.18–0.37), *n* = 103	0.74 (0.54–1.01)	0.82 (0.61–1.10)
6 months	0.49 (0.34–0.71), *n* = 92	0.26 (0.17–0.39), *n* = 94	0.96 (0.72–1.29)	1.08 (0.81–1.43)
1 year	0.43 (0.27–0.66), *n* = 84	0.20 (0.13, 0.30), 92	0.66 (0.49–0.89)	0.75 (0.57–0.98)
FSH (IU/ml)				
Baseline	5.48 (3.90–7.71), *n* = 41	5.65 (4.04–7.90), *n* = 38		
6 weeks	6.45 (5.31–7.82), *n* = 35	8.27 (6.31–10.83), *n* = 37	1.20 (0.86–1.67)	1.11 (0.79–1.54)
6 months	6.41 (4.85–8.46), *n* = 34	7.37 (4.84–11.21), *n* = 35	1.14 (0.70–1.84)	1.03 (0.64–1.65)
1 year	7.90 (5.66–11.04), *n* = 36	10.80 (6.74–17.29), *n* = 34	1.38 (0.80–2.39)	1.26 (0.74–2.13)
LH (IU/ml)				
Baseline	5.26 (3.70–7.46), *n* = 41	5.09 (3.64–7.13), *n* = 38		
6 weeks	7.05 (5.38–9.23), *n* = 35	5.91 (3.83–9.14), *n* = 37	0.82 (0.51–1.34)	0.80 (0.49–1.29)
6 months	5.79 (4.45–7.53), *n* = 34	6.90 (4.56–0.45), *n* = 35	1.22 (0.76–1.96)	1.16 (0.73–1.86)
1 year	7.69 (5.43–0.90), *n* = 36	7.42 (4.67–11.78), *n* = 34	0.95 (0.53–1.67)	0.91 (0.52–1.59)

*Note*: None of these results reach statistical significance. Abbreviations: AMH, anti‐Müllerian hormone; CI, confidence interval; FSH, follicle‐stimulating hormone; LH, luteinizing hormone; UAE, uterine artery embolization.

^a^ Values are given as geometric mean (95% CI).

^b^ Estimates >0 favor myomectomy.

### 3.5 Ovarian reserve

Hormone assay data are reported as geometrical means, unadjusted and adjusted for baseline scores and also age. There was no significant difference between the levels of hormones associated with ovarian reserve in each group.

## 4 DISCUSSION

While both procedures improved participant‐reported health‐related QOL scores, women assigned to the myomectomy group reported higher scores than those in the UAE group. The menstrual bleeding scores appeared similar in both groups. The overall complication rates for both procedures were low. The need for additional treatments was higher in the UAE group, and length of hospital stay was shorter in the UAE group. There were no consistent differences between the groups in biomarkers of ovarian reserve, and too few pregnancies in the present study to conclude the effect of the procedures on fertility.

The doubling of the UFS‐QOL score from baseline to each time point shows that both treatments are effective, with some additional benefit accrued from myomectomy equating to a small to moderate standardized treatment benefit at 2 years.^17^

There were substantially more surgical re‐interventions in the UAE group within 2 years of follow‐up, possibly reflecting the higher residual impact on QOL observed in the UAE group and the marginal patient‐reported preference for myomectomy.

Similar large improvements in UFS‐QOL scores have been reported after various fibroid treatments,^21^, ^22^ although the FEMME trial is unique in its robust comparison of myomectomy and UAE.

Previous randomized trials that measured FSH, and used varying thresholds for ovarian failure, also found no evidence of harm from UAE over short and longer time frames.^23^

The present study is the largest ever randomized clinical trial to report on the treatment of symptomatic fibroids by UAE and any surgery. The robust study design ensures internal validity, enabling the results to be interpreted with confidence. In addition, the other measures reported above reflect the aspects of the condition of most importance to women.

The techniques used for both myomectomy and UAE were determined by the fibroid presentation and the preference of the operating clinician. This enabled a diverse range of fibroid diagnoses to be included in the study. There was an 81% response rate for the primary outcome.

There were a substantial number of women who were not recruited due to their preference for a particular treatment option, and the expectations of treatment benefit were not captured before randomization. All participants were analyzed in the groups to which they were allocated and the per‐protocol analysis gave a treatment effect very similar to the ITT population analysis, suggesting little impact of non‐adherence to the allocation.

The two procedures have considerably different recovery periods, which may be reflected in the first outcome measures reported by participants at 6 months. The mean duration from randomization to the procedure was approximately 13 weeks in both groups, and the primary outcome was to compare QOL at 2 years postoperatively as a realistic time point.

The present trial did not select patients based on their pregnancy intentions. The generalizability of the findings is increased by the inclusion of multiple centers, gynecological surgeons, and interventional radiologists. This allowed for the evaluation of the impact of both interventions without confounding by individual variance in clinical practice and skill, although nearly two‐thirds of participants were recruited from just three hospitals.

Some blood samples were not analyzed as FSH and luteinizing hormone were not obtained within 5 days of the start of the last menstrual period. Despite randomization, there was a small difference in the age of participants between the two groups, prompting a post‐hoc adjusted analysis of the ovarian reserve markers.

The data from the present study showed that myomectomy was cost‐effective in the UK healthcare setting^24^; however, this does not take into account that UAE is less invasive. Given that both procedures are clinically effective, UAE may be more attractive in low‐resource settings as it requires a shorter hospital stay (median 4 days compared with 2 days) and has the potential to be completed as an outpatient procedure. In the present study, no immediate/pre‐discharge infection was observed in the UAE group, whereas this was 4% in the myomectomy group. Post‐discharge complications were recorded, from discharge to 6 weeks after discharge, and showed infection rates of 14% in the UAE group and 17% in the myomectomy group.

UAE is now an established treatment for symptomatic fibroids but is mainly used in high‐income countries. The uptake in low‐income countries has been slow.^25^ The life‐saving benefits of UAE in obstetrics (i.e., major obstetric hemorrhage) would be another incentive for its use in low‐resource settings. As Lessne et al.^26^ suggested, carefully planned resource allocation, training, and selection of patients are crucial in maintaining a safe and sustainable interventional radiology service in low‐ and middle‐income countries (LMICs). A 2011 report from Brazil^27^ demonstrated an excellent example of a novel adaptation on UAE in low‐resource settings.

The present study can form a solid evidence base in LMIC settings to support the studies arising from the regions themselves.

In conclusion, both UAE and myomectomy are effective treatments for improving the QOL of women with symptomatic uterine fibroids. They both decrease the incidence of heavy menstrual bleeding equally. Neither treatment impacted significantly on measures of ovarian reserve.

Myomectomy and UAE are both established procedures within the repertoire of many gynecologists and interventional radiologists, respectively, and the training of junior doctors should continue to include these procedures. Services should continue to offer both procedures to women where both are potential options. Women should be provided with the evidence generated by the FEMME trial to enable them to make a fully informed decision regarding their fibroid treatment.

## AUTHOR CONTRIBUTIONS

Fusun Sirkeci: investigation, writing (review and editing). Jane Daniels: conceptualization, writing (original draft), writing (review and editing), project administration, investigation, funding acquisition. Lee Middleton: statistical analysis plan, data curation, formal analysis, writing (review and editing). Versha Cheed: data curation, formal analysis, writing (review and editing). Isaac Manyonda: conceptualization, site principal investigator, writing (review and editing), investigation, funding acquisition. Anna M. Belli: conceptualization, site principal investigator, writing (review and editing), investigation, funding acquisition. John Moss: conceptualization, site principal investigator, writing (review and editing), investigation, funding acquisition. Olivia Wu: conceptualization, writing (review and editing), funding acquisition. Klim McPherson: conceptualization, statistical analysis plan, writing (review and editing), funding acquisition. Mary A. Lumsden: conceptualization, site principal investigator, writing (review and editing), investigation, funding acquisition.

## FUNDING INFORMATION

The present study was funded by the UK National Institute of Health Research (NIHR) Health Technology Assessment (HTA) program (project number 08/53/22).

## CONFLICT OF INTEREST

MAL reports personal fees from Gedeon Richter outside the submitted work (2018). All other authors declare they have no competing interests. All authors have completed the unified competing interest form at www.icmje.org/coi_disclosure.pdf (available on request from the corresponding author) and declare (1) no financial support for the submitted work from anyone other than their employer; (2) no financial relationships with commercial entities that might have an interest in the submitted work; (3) no spouses, partners, or children with relationships to commercial entities that might have an interest in the submitted work; and (4) no non‐financial interests that may be relevant to the submitted work.

## TRIAL REGISTRATION

https://www.isrctn.com/ISRCTN70772394.

## Data Availability

Research data are not shared.
